# A Short-Form Measure of Loneliness to Predict Depression Symptoms Among Adolescents

**DOI:** 10.1007/s10578-022-01370-3

**Published:** 2022-05-27

**Authors:** Michael J. Kyron, Stephen Houghton, David Lawrence, Andrew C. Page, Simon C. Hunter, Sashya Gunasekera

**Affiliations:** 1 Graduate School of Education, University of Western Australia, Perth, WA 6009 Australia; 2 School of Psychological Science, University of Western Australia, Perth, WA Australia; 3 Department of Psychology, Glasgow Caledonian University, Glasgow, Scotland

**Keywords:** Loneliness, Depressive symptoms, Adolescents, Predicting

## Abstract

The purpose of this study was to produce a short-form measure of loneliness and assesses its prediction of depressive symptoms relative to a comprehensive measure. Western Australian adolescents completed the Friendship Related Loneliness and Isolation subscales of the Perth Aloneness Scale (PALs) three times over 18 months (T 1 *n* = 1538; T 2, *n* = 1683; T 3, *n* = 1406). Items were reduced while preserving predictability. Follow-up confirmatory factor analyses and predictive models with the reduced and full PALs were then tested. A reduced six-item scale (PALs-6) preserved the two-factor structure of the PALs and showed strong prediction of very elevated depressive symptoms (Sensitivity = 0.70, Specificity = 0.78, AUC = 0.81); it was less successful in predicting future symptoms (Sensitivity = 0.67, Specificity = 0.64, AUC = 0.74). The PALs-6 provides a brief measure of adolescent loneliness for clinicians and researchers that also predicts very elevated levels of depression.

**Supplementary Information:**

The online version contains supplementary material available at 10.1007/s10578-022-01370-3.

## Introduction

Depression is a common and often devastating psychological disorder that is one of the top five leading causes of disability and disease burden worldwide [1, 2]. Early adolescence is a critical developmental period for the emergence of depression [3], with research suggesting that overall levels of depressive symptoms increase markedly over the course of this period [4, 5]. According to aggregated published data, the probability of depressive disorders increases from 5% in early adolescence to 20% by the end of this age period [6]. Moreover, onset of depression in adolescence confers a specific high risk for chronic recurrence and poor functioning throughout the lifespan [7, 8], including poor psychosocial functioning [9], cognitive impairment [10], and even suicidality [11].

Depression typically leads individuals to become isolated from family and friends and although loneliness has often been identified as an important risk and predictive factor of adolescent depression [12], the impact of loneliness on the health and development of young people is only now being understood [13]. Predicted to reach epidemic proportions by 2030 [14], loneliness is defined as a distressing emotional state people experience when they notice a discrepancy between the desired and perceived quality or quantity of their social relations [15, 16]. The research is unequivocal that loneliness is linked with a constellation of mental health problems (e.g., depression, anxiety: [17–24]) and has negative impacts on physical wellbeing (e.g., somatic complaints, sleep problems: [25, 26]). A trajectory of increasing loneliness during adolescence predicts future depression, self-harming, and suicide ideation [27, 28].

Recent research [29] has examined risk factors for suicidal behaviours using a network modelling approach (i.e., where mental health problems are viewed as a system where symptoms form interacting networks among themselves and give rise to each other as epiphenomena as the diseases progress) [30]. In this research, items from the Children’s Depression Inventory (CDI) [31] completed by a community sample of approximately 1400 13–19 year olds, identified loneliness as a key central node in the network of symptoms. In a replication study with 5888 12–16 year olds [32], loneliness and sadness were the most central symptoms in a network of adolescent depression symptoms, with loneliness explaining the most variance of suicide ideation.

There is strong evidence that the developmental course of loneliness is heterogeneous [33], and is best represented by a multidimensional model, varying in intensity and across causes and circumstances, and where different social relationships give rise to different forms of loneliness [16, 34–37]. It is also known that loneliness can fluctuate in daily life, depending on, among others, the context people are in [38, 39]. Only one study seems to have examined the temporal dynamics of adolescent loneliness, however [40]. Although the authors reported not using “stringent statistical tests” (p. 926) the data gathered revealed that levels of adolescent’s loneliness varied over time according to the contexts, situations, and company they were in at that time.

Like loneliness, depressive symptoms can also vary [41] and fluctuations in affective states can signify issues with emotional regulation and exhibit proximal relationships with short-term spikes in suicidal ideation and self-harm [42]. Identifying adolescents at-risk of experiencing psychological distress is inhibited by such fluctuations in affective states and associated risk/protective factors over short-term periods. Although temporal stability may be evident over longer periods there are periods of imbalance over short-term periods that signify risk for adverse outcomes (e.g., psychological distress, substance use, and self-injury). Long time periods between assessments may therefore fail to accurately identify symptoms and obscure associations with predictors.

Aided by advances in mobile technology recent research has focused on the intensive assessment of psychological risk and protective factors to enhance understanding and detection of adverse states. For instance, clinical patients were assessed several times daily using mobile ecological momentary assessment (EMA) software, and nearly all were found to have dramatic changes in suicidal ideation and loneliness over a single day [43]. Compared with healthy controls, depressed individuals tend to report greater instability and reactivity to positive events in prior research [44]. Negative fluctuations in affective states over short-term periods (i.e., hours, days) have been linked to an increased propensity to desire suicide and engage in self-injurious behaviours [45, 46]. A recent meta-analysis [47] examined the associations between daily interactions and negative affect, finding perceptions of social interactions to be associated with within-person fluctuations in positive and negative affect. Other research has found adverse interpersonal states, such as thwarted belongingness (i.e., low perceived support, isolation), to be associated with suicidal ideation [48].

Given the strong associations between loneliness, broader interpersonal states and aspects of mood, clinicians and researchers would benefit from a brief, multi-dimensional measure of loneliness with predictive capabilities. This is not to say that current measures to evaluate loneliness do not already exist. A recent review [49] identified the most commonly used instruments as the UCLA Loneliness Scale (UCLA) [50] and its shortened 11‐item [51], 8‐item [52, 53], 6‐item [54, 55] and 3‐item versions [56]. Theoretically, while these successive versions have conceptualized loneliness as a single dimension, the factor structure has varied between one and three factor solutions. Other measures identified in the review included the Children’s Loneliness and Social Dissatisfaction Scale [57] and the Loneliness and Aloneness Scale for Children and Adolescents ([58]. However, all these measures were developed some time ago [49] and “each omitted key processes, including interviews with children and adolescents” [49 p. 11] suggesting that their views of the loneliness experience did not inform the measures.

A promising measure of adolescent loneliness that did include the voices of adolescents during its development [59] is the Perth A-loneness scale (PALs, 37). The PALs comprises four distinct factors: friendship-related loneliness, isolation, negative attitude to being alone, and positive attitude to being alone. A series of studies [37, 60, 61] has consistently reported satisfactory fit statistics of a four-factor model and internal reliability. Test–retest reliability (9 months apart) also supports a degree of temporal stability (*rs* = 0.59–0.67) and a recent Rasch analysis of the PALs [62] supported the interval scale measurement properties of the PALs. Of the PALs four factors, friendship-related loneliness (i.e., having reliable, trustworthy supportive friends e.g., *“I can turn to my friends for help when I need it”*) and isolation (i.e., having few friends or believing that there was no one around offering support e.g., “*I feel like I do not have a friend in the world*”) have exhibited particularly strong cross-sectional associations with positive mental wellbeing [60] and depression symptoms [61].

Therefore, the current study explores the effectiveness of a multidimensional measure of loneliness in predicting current and future symptoms of depression. Developing an abbreviated version of the PALs scale, which shows strong connections to depressive symptoms, is beneficial to both comprehensive long-term longitudinal (i.e., monthly, yearly) and intensive research (e.g., daily diary, ecological momentary assessment), and minimises unnecessary burden on participants.

## Materials and Methods

### Participants and Procedure

The school principals of 12 randomly selected schools (ten state government schools and two non-government schools) in Perth, Western Australia were contacted via telephone to ascertain their interest in participating in the research. These schools were located across a range of socio-economic status areas as indicated by their Index of Community Socio-Educational Advantage (ICSEA). ICSEA is set at an average of 1000 (SD = 100) and the higher the ICSEA value, the higher the level of educational advantage of students who go to this school (and vice versa). The ICSEA values ranged from 904 to 1191.

All of the school principals agreed to participate and information sheets explaining the research, along with consent forms for parents, were subsequently delivered to the schools. The information sheets were distributed to students in school grades 5 (10 years of age) to 9 (up to 15 years of age). Informed consent was obtained from individual participants who were assessed via an electronic survey on three separate occasions over the span of approximately 18 months as they progressed through their school grade levels. The lag between Time 1 and Time 2 was 6 months, while there was a longer lag from Time 2 to Time 3 of 12 months.

Each participant received a unique four-digit identification code, which allowed them to log on to the survey. This unique code ensured that all information provided was confidential and that all participants’ data could be linked to future administrations. To ensure measures were administered consistently across schools, one teacher in each of the schools volunteered to be responsible for liaising with the researchers and administering the survey. Written instructions regarding administration procedures were provided to all of these teachers along with verbal instructions. The measures were completed during regular school hours and a teacher and/or school psychologist was present to support any students who had difficulty understanding any items. The electronic survey remained open for approximately four weeks.

At Time 1, the total sample comprised of 1538 adolescents. Participation across the following two assessment points remained high (Time 2, *n* = 1,683; Time 3, *n* = 1,406). Around 81.2% of participants at Time 1 completed assessments at Time 2, and 50.2% completed Time 3. The cohort at Time 2 was larger due to additional schools joining the study. Of the sample, 18.9% were diagnosed by a paediatrician or child psychiatrist as having a neurodevelopmental disorder (NDD). This was established by asking students to self-report a formal NDD diagnosis. The accuracy of this was subsequently confirmed by the school principal and/or school psychologist who matched participant’s four figure unique codes to a master list of participants’ names. The full demographic characteristics at each time point have been outlined in Table 1.

**Table 1:** Sample characteristics

		6-month Lag	1-year Lag
	Time 1 (*N*)	Time 2 (*N*)	Time 3 (*N*)
Total	1538	1683	1406
Remaining from T1	–	1237 (81.2%)	765 (50.2%)
Remaining from T2	–	–	870 (52.1%)
Sex			
Male	641 (41.7%)	723 (43%)	591 (42%)
Female	882 (57.3%)	947 (56.3%)	794 (56.5%)
Other	15 (1%)	13 (0.8%)	21 (1.5%)
School year			
5	173 (11.4%)	3 (0.2%)	1 (0.1%)
6	200 (13.2%)	183 (11%)	3 (0.2%)
7	438 (28.8%)	175 (10.5%)	126 (9.1%)
8	360 (23.7%)	474 (28.5%)	141 (10.2%)
9	328 (21.6%)	396 (23.8%)	401 (29.1%)
10	9 (0.6%)	420 (25.2%)	358 (26%)
11	8 (0.5%)	7 (0.4%)	341 (24.7%)
12	3 (0.2%)	6 (0.4%)	8 (0.6%)
Age			
10	118 (7.8%)	7 (0.4%)	3 (0.3%)
11	200 (13.2%)	171 (10.3%)	5 (0.5%)
12	311 (20.5%)	179 (10.7%)	99 (9.4%)
13	408 (26.9%)	440 (26.4%)	156 (14.9%)
14	325 (21.4%)	412 (24.7%)	352 (33.5%)
15	144 (9.5%)	406 (24.4%)	376 (35.8%)
16	10 (0.7%)	47 (2.8%)	54 (5.1%)
17	3 (0.2%)	5 (0.3%)	5 (0.5%)
CDI category			
Low	819 (54.4%)	943 (56.6%)	725 (53.2%)
Moderate	172 (11.4%)	198 (11.9%)	175 (12.8%)
Elevated	146 (9.7%)	142 (8.5%)	134 (9.8%)
Very elevated	368 (24.5%)	383 (23%)	328 (24.1%)

Permission to conduct this research was obtained from the Human Research Ethics Committee of the administering institution, the State Department of Education, and the principals of all schools. Permission was also granted by the publisher of the CDI 2: SR[S] to administer the instrument online.

### Measures

**Friendship Related Loneliness and Isolation** The Perth A-loneness scale is a validated 24-item self-report measure of adolescent loneliness, comprising four correlated factors, each with six items [37]. Factor One measures quality of friendships (e.g., “*My friends will stand by me in almost any difficulty*”); Factor Two, feelings of isolation (e.g., “*I feel like I do not have a friend in the world*”); Factor Three, positive attitudes towards being alone (e.g., “*I have discovered the benefits of being alone*”); and Factor Four, negative attitudes towards being alone (e.g., “*When I am all by myself, I wish I had a friend to be with*”). Participants respond using a six-point Likert scale: 1 = never, 2 = rarely, 3 = sometimes, 4 = often, 5 = very often, 6 = always. Given the stronger correlations between depression symptoms and both friendship related loneliness and isolation subscales, the current study focused on both scales for use in predicting current and future symptoms. The friendships (Time 1, α = 0.90; Time 2, α = 0.90; Time 3, α = 0.91) and isolation scales (Time 1, α = 0.83; Time 2, α = 0.85; Time 3, α = 0.87) showed good internal consistencies.

**Children’s Depression Inventory – 2** (self-report short version; CDI 2: SR[S]) [31] is a brief self-report assessment of cognitive, affective and behavioural symptoms of depression in children and adolescents aged 7–17 years. The CDI 2: SR[S] comprises of 12 items, each with three separate sentence response options that describe participants’ feelings and ideas over the past 2 weeks. Each item is measured on a 3-point Likert scale, with higher scores indicating poorer outcomes (e.g., 0 = I am sad once in a while, 1 = I am sad many times, 2 = I am sad all the time). The CDI 2: SR[S] has demonstrated good reliability, and discriminant and convergent validity in prior research [61]. In the present study, the CDI 2: SR[S] had good internal consistency at Time 1 (α = 0.87), Time 2 (α = 0.85), and Time 3 (α = 0.87). Standardized CDI scores can be categorized based on the number of standard deviations from the mean. In the current study, the focus will be on “very elevated” depression symptoms, which represents two standard deviations from the mean (23–24% of the sample from Time 1 to Time 3).

### Statistical Approach

Item reduction was conducted in line with Koczkodaj [63], which outlines an approach to reducing the number of scale items without losing predictability. The approach examines the individual classification of respective items of a scale. Specifically, the area under curve statistics are calculated for individual variables, which are arranged in ascending order. The attribute with the largest AUC is sequentially added to a subset of attributes with the next largest AUC, creating a running total. If the AUC characteristic decreases, then the procedure stops due to a suggested lack of benefit for additional items in improving prediction. The *RatingScaleReduction* package in R was used to calculate optimal number of items (while preserving prediction) based on cross-sectional and prospective prediction. Items were selected based on consistent cross-sectional and prospective prediction. Further, any repetitious items that exhibited overlap with other items were excluded from the shortened scale.

A follow-up confirmatory factor analysis was conducted using MPlus to examine the construct validity of the one and two factor models for the brief loneliness measure. Model fit was assessed based on guidelines by Kenny [64], with a Root Mean Square Error of Approximation (RMSEA) below 0.08, and a Comparative Fit Index (CFI) and Tucker Lewis Index (TLI) greater than 0.9, indicating good model fit.

The performance of the two reduced scales in predicting very elevated depression symptoms was assessed using logistic regression. Repeated cross-validation was used to produce a less biased model on 70% of the training data and tested on a smaller test portion of data (30%). Statistics that assess prediction performance can be subject to pre-determined probability for classification of an event. Probability was adjusted to detect 70% of adolescents with very elevated depression on the training data set, with this cut-off applied to the test portion. The remaining statistics were assessed based on these cut-offs, including specificity (i.e., accurately classifying adolescents who do not have very elevated depression), positive predictive value (i.e., proportion of accurately identified cases to false-positives), and negative predictive value (i.e., proportion of adolescents predicted not to have very elevated depression compared to false negatives).

**Missing data.** In total, 52% of data were missing at some time point, with 22% completing only one time point. Model performance was initially assessed using casewise exclusion given the large amount of available data, complexity in estimating individual scale-item scores, and long lags between assessments. For the 2103 participants across all three time points, there were 4520 cross-sectional assessment points and 3163 prospective assessment points (i.e., 2 consecutive completed assessments). Model performance was also assessed when CDI 2: SR[S] and PALs total scores, and scores from the shortened version of the PALs developed in the current study, were imputed using k-nearest neighbour algorithms. This approach selects the closest observations (neighbours) according to a distance metric, where the selected observations present known values on the features to be imputed. A weighted average of these values is then used as an estimate for each incomplete feature value [65]. Prospective prediction was found to be almost identical, while cross-sectional prediction was lower on the imputed dataset (Supplementary Table 1). For this analysis, there were 6285 cross-sectional and lagged assessment points for the 2103 participants after imputation.

Little’s Missing Completely At Random test [66] was performed using SPSS software. The MCAR criteria was satisfied at Time 1 (χ^2^ = 2.992, DF = 5, Sig. = 0.701), Time 2 (χ^2^ = 4.113, DF = 3, Sig. = 0.250), and Time 3 (χ^2^ = 2.016, DF = 2, Sig. = 0.365). Logistic regressions were run to assess whether particular factors predicted non-participation at Time 2 and Time 3. Depression symptoms and both friendship related loneliness and isolation did not significantly predict future participation. Further, there were no significant differences in terms of gender and NDD diagnosis with regard to missing data. However, adolescents 13 years of age at Time 1 were statistically less likely to participate at Times 2 and 3 than other age groups. Therefore, participation in the survey which aimed to measure depression and loneliness symptoms appeared unrelated to these specific factors and thus fulfilled missing at random assumptions [67].

## Results

### Item Selection

**Fig. 1:**
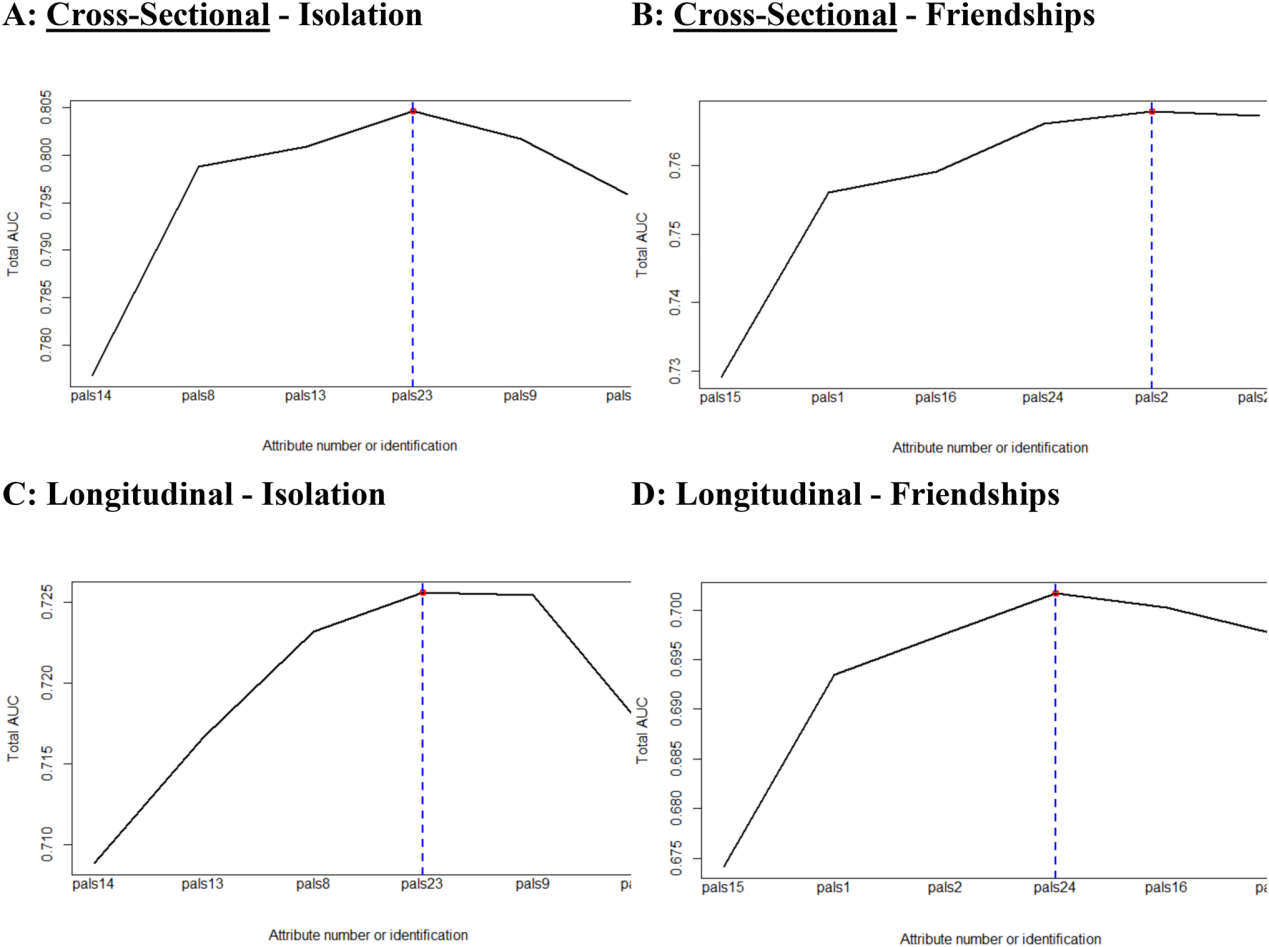
The cumulative AUC statistics for the friendship and isolation related loneliness subscale items in predicting current and future very elevated depression symptoms

The prediction of very elevated depression symptoms of the individual PALs scale items, and cumulative increase in prediction is displayed in Fig. 1. For the Isolation subscale, the top three items associated with increased AUC metrics both cross-sectionally and longitudinally were PALs item 8 (“*I feel like I do not have a friend in the world*”), PALs item 13 (“*I have nobody to talk to*”), and PALs item 14 (“*No one cares much about me*”).

For the Friendship Related Loneliness (i.e., quality of friendships) subscale, four items were associated with increases in AUC metrics both cross-sectionally and longitudinally: PALs item 15 (“*I get plenty of help and support from friends*”), PALs item 1 (“*I feel part of a group of friends*”), PALs item 24 (“*Most of my friends are true friends*”), and PALs item 2 (“*I can turn to my friends for help when I need it*”). With an aim of developing a sufficiently shortened scale that evenly captures both friendship related loneliness and isolation, the PALs item 2 (“*I can turn to my friends for help when I need it*”) was not selected based on the overlap with PALs item 15. That is, both the PALs item 2 and PALs item 15 measured support from peers, with the correlation between both items being high (*r* = 0.66, *p* < 0.001). Supplementary Table 2 shows the full PALs and PALs 6 items.

### Factor Analysis

**Fig. 2:**
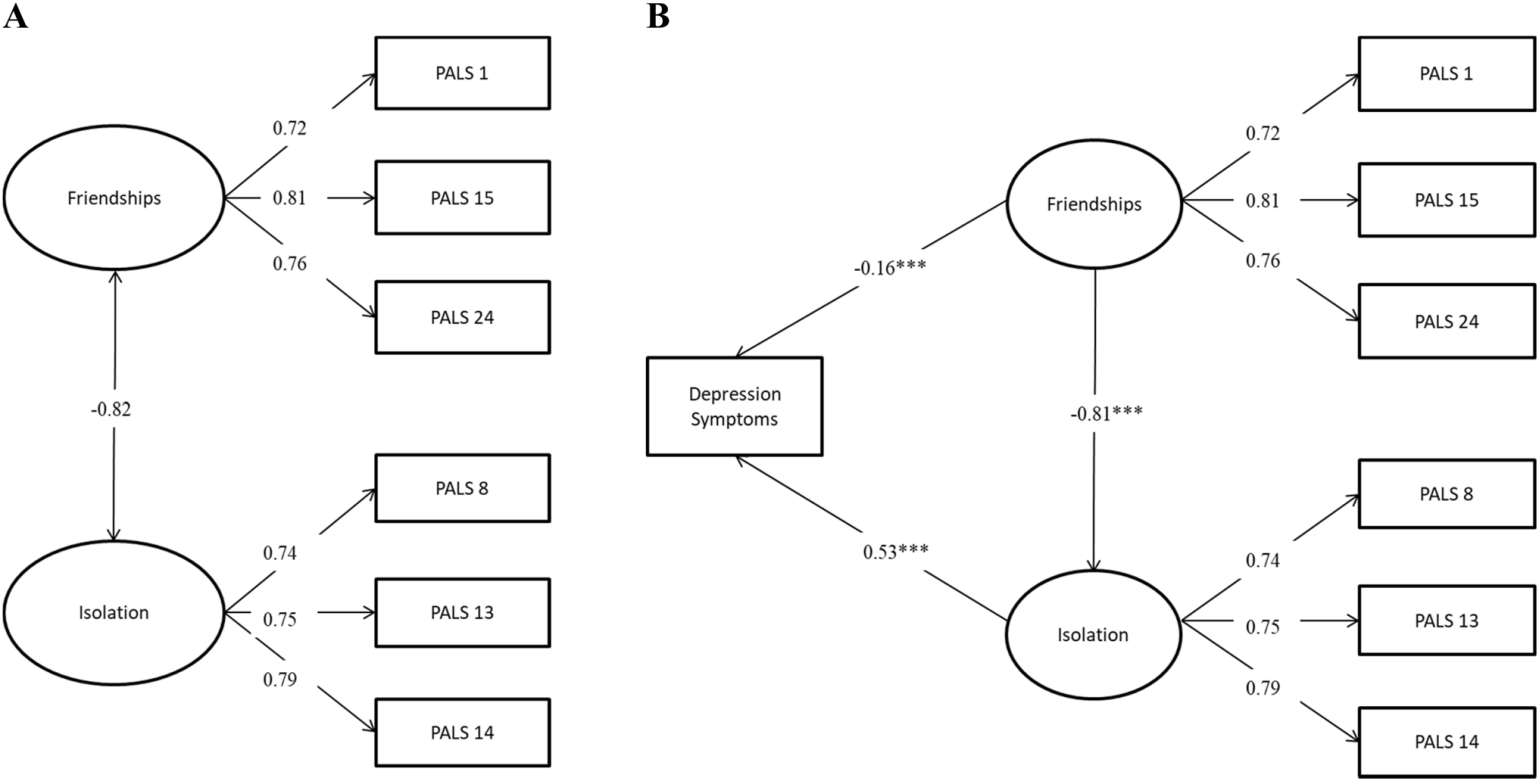
Confirmatory factor analysis for the reduced-item PALs-6 scale (Panel A). Associations with depression are cross-sectional (Panel B)

A confirmatory factor analysis was conducted to assess the fit of a one factor and two-factor model to the data (Fig. 2A). A one-factor model had relatively poor fit to the data, with a high RMSEA 0.14 (0.13–0.15), and relatively low TLI (0.88) and CFI (0.93). On the other hand, the two-factor model exhibited good fit, with low RMSEA (0.06, 95% CI 0.06, 0.07) and high CFI (0.99) and TLI (0.98) statistics. The follow-up structural equation model also had good fit to the data (Fig. 2B; RMSEA = 0.06, 95% CI 0.05–0.07, CFI = 0.99, TLI = 0.98). Both PALs sub-scales were found to be highly, inversely correlated (*r* =  − 0.82, *p* < 0.001) and both Isolation (B = 9.71, β = 0.53, *p* < 0.001) and Friendship Related Loneliness (B =  − 2.35, β =  − 0.16, *p* < 0.001) subscales were significantly associated with current depression symptoms, accounting for 44% of variance (*p* < 0.001).

### Predictive Performance

**Fig. 3:**
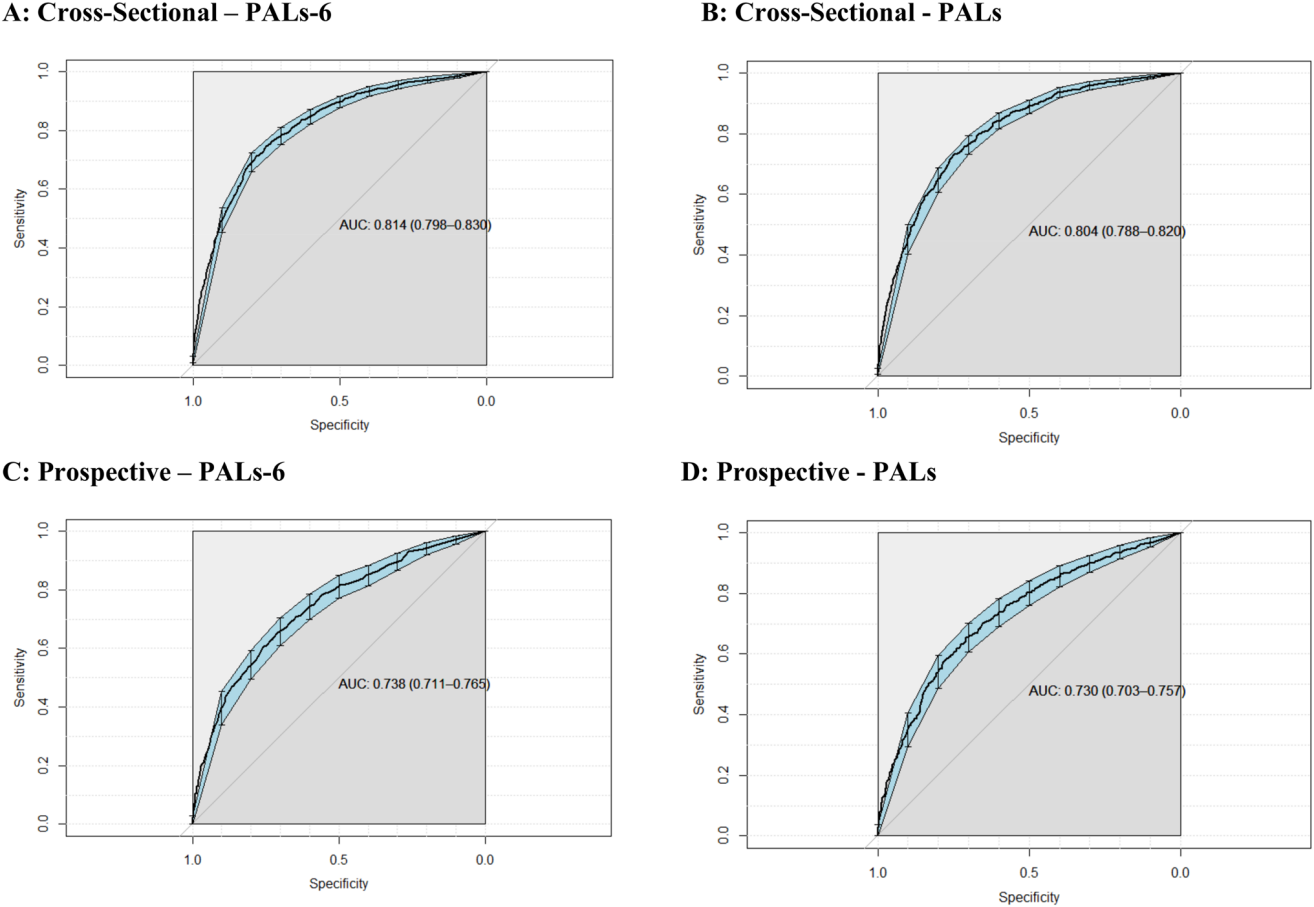
Receiver Operator Curves for brief and full models

The performance of the reduced and full PALs scales in predicting very elevated depression symptoms is presented in Table 2, and ROC curves presented in Fig. 3. The accuracy and area under curve statistics of both models was found to be almost identical, indicating the reduced number of items did not affect the predictive performance of the scale. Both the reduced and full scales had comparable positive predictive value statistics (Reduced Scale = 0.50, Full Scale = 0.48) when applied cross-sectionally, indicating a false-positive in roughly half of all predicted cases. Prospective prediction had lower specificity, positive predictive value, and area under curve statistics for both models.

**Table 2:** Performance of the reduced and full PALS scales in predicting current and future symptoms

	Cross-sectional		Longitudinal	
	PALs-6	PALs	PALs-6	PALs
Model performance				
Sensitivity	0.70	0.72	0.67	0.64
Specificity	0.78	0.76	0.64	0.64
PPV	0.50	0.48	0.36	0.34
NPV	0.89	0.90	0.87	0.86
Accuracy [95% CI]	0.76 [0.74, 0.78]	0.75 [0.73, 0.77]	0.65 [0.61–0.68]	0.64 (0.60–0.67)
AUC [95% CI]	0.81 [0.80, 0.83]	0.80 [0.79, 0.82]	0.74 [0.71–0.77]	0.73 [0.70–0.76]

*PPV* positive predictive value. *NPV* negative predictive value. *AUC* area under curve. Performance metrics based on prediction on the test portion of data (30%). Specification of probability cut-offs for a predicted event based on 70% sensitivity on the training set. *PALs-6* perth adolescent loneliness scale—6-item. *PALs* perth adolescent loneliness scale

## Discussion

Given the known strong associations between loneliness and depressive symptoms and that both constructs can fluctuate, it is important to develop a brief measure of loneliness capable of predicting symptoms of depression in a way comparable to a more comprehensive, well-validated measure. A brief measure of loneliness would also reduce the burden on young people when repeated measurements over time are necessary. Future advances in this area of research are likely to be dependent, in part, on the development of such a brief loneliness measure. A short form can facilitate both intensive research work (such as that using ecological momentary assessment) and more traditional longitudinal work stretching across months or years.

The outcome of the present research, the reduced PALs—6, is a multi-dimensional measure of loneliness which halves participant load to 6 items as compared to the 12 items of the two subscales of the PALs. PALs—6 preserves cross-sectional relationships with extreme depression, though both the PALs and PALS—6 were less successful in longitudinal prediction. The reduced 6-item measure (PALs—6) preserved the two-factor nature of the original two PALs subscales when capturing friendship related loneliness and isolation. Thus, the new brief scale captures both quality of friendships and aspects of isolation to define adolescents’ experiences of loneliness.

Adolescence is known to be a sensitive developmental phase, a time when insufficient connections to others can lead to profound and lasting negative consequences on physical and mental health, even leading to increased mortality [68]. Conversely, quality friendships provide numerous social and emotional benefits [69]. This applies to young people worldwide. Thus, the abbreviated PALs—6 scale is an important contribution to research aiming to assess an important risk factor for current psychopathology and for use in intensive, short-term research.

Cross-sectional prediction of very elevated depression symptoms in the current research was satisfactory, showing a good balance between sensitivity and specificity. On the other hand, longitudinal prediction was associated with higher misclassification rates. This may in part be due to the limitations of classification over long-term periods, particularly with both dependent and independent variables being prone to change over time. For instance, interpersonal factors and depression symptoms are known to fluctuate over a matter of hours or days, and therefore isolated measurement may not capture “typical” patterns in symptoms and risk factors [42]. Interpreting positive predictive value is largely dependent on the outcome in question (e.g., prediction of suicide completion is typically associated with exceptionally high false positive rates of approximately 99%; [70]). Further, the consequences from false negatives (i.e., failing to detect someone with depression) may outweigh the cost of false positives (i.e., incorrectly targeting resources to adolescents not at short-term risk of depression). Inferences regarding an acceptable PPV can be made through comparisons to similar studies predicting depression symptoms. For example, Seeley et al. [71] assessed the predictive quality of several interpersonal related composite scores, including low peer support (PPV = 0.17), low parental support (PPV = 0.22), and poor school functioning (PPV = 0.24). Another community-based study of older adults [72] found a brief measure of loneliness predicted moderate to severe depression symptoms cross-sectionally with a PPV of 0.26. The PPV of the PALs—6 scale is therefore high relative to the few studies which have provided PPV statistics of individual scales when predicting depression symptoms. Improving PPV may be enhanced through repeated administration of the current scale to identify adolescents with persistent loneliness and by assessing a broader range of correlates for depression.

## Limitations

There are limitations to the current study. First, data were self-report and based on reflection. Young people frequently have difficulty reporting their internal states to other sources such as parents and teachers, and parents and teachers have difficulty perceiving the internal world of their children [73]. Therefore, self-report measures may elicit valid responses from young people. Second, predicting depressive symptoms over short-term periods was beyond the scope of the current study, and should be assessed to determine optimal lags between assessments when predicting future symptoms using the PALs—6. Third, there was a notable proportion of missing data over time, although there was no systematic pattern of missing data detected. That is, adolescents with better or worse mental health were not significantly more likely to have missing data. Fourth, there was a high proportion of adolescents who met the criteria for “very elevated” depression symptoms. Approximately 3% of adolescents should be two standard deviations above the normative average [74]. However, some CDI 2: SR[S] items were endorsed a considerably high amount, and may have inflated the number of adolescents in the very elevated range. For instance, some 25% of adolescents endorsed the items “*I have to push myself all the time to do my schoolwork*” and “*I am tired all the time*”. With the original CDI-2 dating back to 1992, there may be important changes in pressures faced by adolescents.

Although we are only now beginning to develop an understanding about adolescent loneliness, the research evidence is unequivocal that it is an important risk factor for current psychopathology. Loneliness is predicted to reach epidemic proportions within the next 10 years and is rightly viewed as a global public health disorder [14]. Following a U-shaped curve over the lifespan, loneliness peaks among adolescents and older adults [75]. For some adolescents, loneliness can create a sense of “paralyzing hopelessness and unutterable futility” [76, p. 7] and can lead to catastrophic outcomes. The research evidence is unequivocal that loneliness is an important risk factor for current psychopathology. Thus, research aimed at using loneliness to predict adverse events in longitudinal research, aided by brief measures that preserve the qualities of a more comprehensive measure, is critical.

## Summary

Although half the length of the PALs, the PALs—6 was almost identical in its ability to predict adolescents with very elevated levels of depression both cross-sectionally and longitudinally. Given that loneliness can fluctuate over time and has strong associations with adverse mental health, the PALs—6 provides clinicians and researchers with a suitable brief measure for identifying young people at risk.

### Supplementary Information

Below is the link to the electronic supplementary material.

Supplementary file1 (DOCX 19 kb)
