# Supplementary material for: A Short-Form Measure of Loneliness to Predict Depression Symptoms Among Adolescents
Source: Child Psychiatry Hum Dev. 2022 May 27;54(6):1760–70. doi: 10.1007/s10578-022-01370-3 (PMC10581951; doi:10.1007/s10578-022-01370-3)
Supplement: Supplementary file 1 — Supplementary file1 (DOCX 19 kb) [file 10578_2022_1370_MOESM1_ESM.docx]

**Supplementary Table 1**

*Correlations between study variables.*

|  | **1.** | **2.** | **3.** | **4.** | ***M*** | ***SD*** | ***Skew*** | ***Kurtosis*** | ***α*** | ***ICC***  ***(6-month)*** | ***ICC***  ***(1 year)*** |
| --- | --- | --- | --- | --- | --- | --- | --- | --- | --- | --- | --- |
| 1. CDI-2 (Continuous) | - | - | - | - | 59.52 | 13.54 | 0.48 | -0.60 | 0.87 | 0.79 | 0.77 |
| 1. Friendship (PALs-6) | -0.44 | - | - | - | 14.32 | 3.15 | -0.82 | 0.07 | 0.83 | 0.69 | 0.63 |
| 1. Friendship (PALs) | -0.45 | 0.81 | - | - | 27.75 | 6.37 | -0.64 | -0.22 | 0.90 | 0.72 | 0.71 |
| 1. Isolation (PALs-6) | 0.49 | -0.63 | -0.58 | - | 5.32 | 2.71 | 1.45 | 2.13 | 0.78 | 0.69 | 0.63 |
| 1. Isolation (PALs) | 0.48 | -0.62 | -0.65 | 0.85 | 10.29 | 4.96 | 1.51 | 2.44 | 0.83 | 0.68 | 0.65 |

*Note*. ICC = Intraclass correlation coefficient. All ICC significant at *p* < .001. α = internal consistency at T1. PALS-6 = PALS – 6-item.

**Supplementary Table 2**

Questionnaire items for the full and brief friendship related loneliness and isolation scales.

*Items in bold comprise PALs - 6

| **PALs and PALs - 6** |
| --- |
| **Friendships** |
| - **PALs 1: I feel part of a group of friends** - PALs 2: I can turn to my friends for help when I need it - **PALs 15: I get plenty of help and support from friends** - PALs 16: My friends will stand by me in almost any difficulty - PALs 22: I have friends that I can trust to do what they say they will do - **PALs 24: Most of my friends are true friends** |
| **Isolation** |
| - **PALs 8: I feel like I do not have a friend in the world** - PALs 9: I am not close to anyone f - **PALs 13: I have nobody to talk to** - **PALs 14: No one cares much about me** - PALs 17: I do not have a close friend - PALs 23: I feel sad because I have no friends |
